# Reviewing the potential of probiotics, prebiotics and synbiotics: advancements in treatment of ulcerative colitis

**DOI:** 10.3389/fcimb.2023.1268041

**Published:** 2023-12-08

**Authors:** Apurva Jadhav, Suresh Jagtap, Suresh Vyavahare, Archana Sharbidre, Bipinraj Kunchiraman

**Affiliations:** ^1^Herbal Medicine, Interactive Research School for Health Affairs, Bharati Vidyapeeth (Deemed to be University), Pune, Maharashtra, India; ^2^Sai Ayurved Medical College, Maharashtra University of Health Sciences, Solapur, Maharashtra, India; ^3^Department of Zoology, Savitribai Phule Pune University, Pune, Maharashtra, India; ^4^Microbial Biotechnology, Rajiv Gandhi Institute of IT & Biotechnology, Bharati Vidyapeeth (Deemed to be University), Pune, Maharashtra, India

**Keywords:** inflammatory bowel diseases, ulcerative colitis, probiotics, prebiotics, synbiotics, therapeutic uses

## Abstract

Inflammatory bowel diseases (IBD) like Crohn’s and ulcerative colitis (UC) are multifactorial pathologies caused by environmental factors and genetic background. UC is a chronic inflammatory disorder that specifically targets the colon, resulting in inflammation. Various chemical interventions, including aminosalicylates, corticosteroids, immunomodulators, and biological therapies, have been extensively employed for the purpose of managing symptoms associated with UC. Nevertheless, it is important to note that these therapeutic interventions may give rise to undesirable consequences, including, but not limited to, the potential for weight gain, fluid retention, and heightened vulnerability to infections. Emerging therapeutic approaches for UC are costly due to their chronic nature. Alternatives like synbiotic therapy, combining prebiotics and probiotics, have gained attention for mitigating dysbiosis in UC patients. Prebiotics promote beneficial bacteria proliferation, while probiotics establish a balanced gut microbiota and regulate immune system functionality. The utilisation of synbiotics has been shown to improve the inflammatory response and promote the resolution of symptoms in individuals with UC through the stimulation of beneficial bacteria growth and the enhancement of intestinal barrier integrity. Hence, this review article aims to explore the potential benefits and underlying reasons for incorporating alternative approaches in the management of UC with studies performed using prebiotics, probiotics, and synbiotics to treat ulcerative colitis and to highlight safety and considerations in UC and future perspectives. This will facilitate the utilisation of novel treatment strategies for the safer and more efficacious management of patients with UC.

## 1 Introduction

Over the past decade, there has been an observed increase in the prevalence and incidence of Inflammatory Bowel Diseases (IBD), specifically Crohn’s Disease (CD) and Ulcerative Colitis (UC) (Molodecky et al., 2012; Roy and Dhaneshwar, 2023). According to a recent epidemiological study conducted by Ng et al. (2017), IBD affects over 0.3% of people in North America and Europe. It is a group of gastrointestinal disorders with chronic inflammation. The cause of IBD is not fully understood but is believed to be a combination of genetic and environmental factors. Factors such as diet, use of antibiotics, and socioeconomic status can influence persistent immune-mediated inflammation in the intestines. (Manichanh et al., 2012; Çavdar and Çavdar, 2023). UC is a chronic gastrointestinal disorder characterised by chronic inflammation of the colorectal mucosa, resulting in non-specific inflammatory changes. The aetiology of this condition remains uncertain, and its conversion into cancer is not easily treatable. Ungaro et al. (2017) conducted epidemiological research which substantiated the global increase in the prevalence of UC. It is a condition caused by a combination of genetic, immune, microbial, and environmental factors. It involves persistent inflammation of the mucosal lining, affecting the rectum and proximal colon segments. The exact mechanisms behind UC development are not fully understood, but it is widely recognised that environmental factors, host genetics, a dysregulated immune system, and microbial dysbiosis play crucial roles. (Ananthakrishnan, 2015).

According to the study conducted by Higuchi et al. (2012), there is evidence to suggest that the adoption of Westernised diets and lifestyles can heighten the susceptibility of genetically predisposed individuals to IBD. Environmental factors encompass various aspects, such as residing in industrialised nations, urbanised settings, and regions situated at higher latitudes. In addition to infections and smoking, other factors also play a role in the pathogenesis of IBD. Patients frequently need lifelong medication, which is the main purpose of therapy, both to persuade clinical remission and to conserve it for a long period. According to the Global Burden of Disease Study, the estimated global prevalence of Crohn’s disease was approximately 6.8 million individuals, while ulcerative colitis affected approximately 10 million individuals. (Alatab et al., 2020).

These data suggest that, in addition to finding effective therapeutic approaches to advance IBD patients’ quality of life and lower healthcare system costs, it is crucial to better understand the factors involved in the development of IBD. Costs of IBD management have increased significantly in the past five years due to different therapeutic interventions and disease characteristics. Patients face increased healthcare utilisation, out-of-pocket expenses, and reduced productivity, necessitating the implementation of cost-effective solutions. (Park et al., 2020) Recent research suggests that microbiota can be therapeutically effective in treating IBD. The gut microbiome influences host processes and can mitigate inflammation. Various therapeutic approaches, including prebiotics, probiotics, and synbiotics, have been developed to alter and restructure the gut microbiome. (Britton et al., 2021) The most common strains currently available as probiotics and possessing beneficial health effects are *Enterococcus faecium*, *Bifidobacterium*, *Bacillus*, *Saccharomyces boulardii (S. boulardii)* and *Lactobacillus strains.* Prebiotics like fructo-oligosaccharides (FOS), isomalto-oligosaccharides (IMO), and xylooligosaccharide (XOS) are studied for their potential benefits on stool volume, constipation relief, and faecal acidity, with easily metabolised by *Bifidobacteria* and other microorganisms. (Guarino et al., 2020).

## 2 Understanding ulcerative colitis

Ulcerative colitis is a chronic inflammatory disorder of the gastrointestinal tract characterised by a progressive decline in health. UC is characterised by inflammation of the mucosal lining, typically confined to the colon and rectum. On the other hand, CD involves inflammation that extends through the entire thickness of the gastrointestinal tract, with a predilection for the ileum, terminal ileum, and colon. The CD is often associated with additional inflammatory conditions outside of the intestines. According to Çavdar and Çavdar (2023), the medical characteristics encompass symptoms such as hemorrhagic diarrhoea, weight loss, fatigue, and abdominal pain. Additionally, certain individuals may develop extra-intestinal manifestations associated with IBD, such as primary sclerosing cholangitis, skin lesions, or joint complications (Judge and Lichtenstein, 2001). The impact of UC on a global scale has been evaluated, revealing that it affects a population of approximately 5 million individuals. UC is characterised by recurring episodes of inflammation in the mucosal layer of the colon, which alternate between periods of relapse and remission. In ulcerative colitis, inflammation is typically confined to the mucosal surface. The disorder originates in the rectum and typically progresses proximally throughout the entirety of the colon. To date, a conclusive therapeutic approach for UC remains elusive, and the currently available interventions primarily focus on sustaining remission (Roy and Dhaneshwar, 2023).

Conventional therapies for UC consist of pharmacological interventions involving the use of various agents, including azathioprine, aminosalicylates, and corticosteroids. These treatments aim to induce remission, prevent relapses, promote mucosal healing, and ultimately reduce the need for colectomy in individuals diagnosed with UC. In recent times, advancements in medical treatments have been influenced by the utilisation of agents such as monoclonal antibodies that specifically target proinflammatory cytokines, adhesion molecules, T-cell activation, and agents that promote the production of anti-inflammatory cytokines, such as IL-10 (Interleukin) and Transforming Growth Factor (TGF). However, the administration of these medications in the context of ulcerative colitis is associated with a distinct set of systemic and localised adverse effects experienced by individuals. These include but are not limited to, symptoms such as headache, abdominal pain, nausea, cramping, loss of appetite, vomiting, and rash. In addition, it is not recommended to use corticosteroids for an extended period due to their potential to induce glucose intolerance, osteoporosis, myopathy, and increased vulnerability to infections. These adverse effects raise significant concerns regarding the safety profile of corticosteroid usage (Mowat et al., 2011). Similarly, the expenses associated with these promising therapeutic approaches are substantial, considering the increasing prevalence and persistent nature of IBD. Histological examinations conducted on patients with UC have revealed the presence of impaired intestinal epithelial barrier function. However, the significance of this dysfunction with the pathology of UC remains uncertain. Patients diagnosed with UC exhibit changes in the function of the epithelial barrier, impaired function of tight junctions, and reduced secretion of mucin-2. Nevertheless, the exact relationship between these abnormalities and chronic inflammation, accompanied by heightened cytokine production, remains uncertain. Heller et al. (2005) conducted a study that yielded evidence indicating that the overexpression of IL-13 plays a pivotal role in the disruption of tight junctions within the intestinal mucosal layer of individuals who have been diagnosed with active UC. The dysfunction of the barrier function in individuals diagnosed with UC has been linked to a heightened absorption of luminal antigens and an elevated susceptibility to bacterial infiltration in the underlying mucosa. Extensive research has provided comprehensive insights into the dysregulation of the intestinal immune system in individuals afflicted with UC. Dendritic cells (DCs) are known to have a pivotal role in the augmentation of UC patients, thus signifying the substantial contribution of these cells in the initiation and perpetuation of inflammatory processes (Hart et al., 2005).

## 3 Conventional treatments for UC

Conventional therapies play a pivotal role in the management of ulcerative colitis and the mitigation of associated symptoms. The objective of these treatments is to manage inflammation, initiate and sustain remission, and enhance the overall quality of life for patients. The Selecting Therapeutic Targets in IBD (STRIDE)-II statement was revised by the International Organisation for the Study of IBD to enhance the treatment of IBD in adults and children. The revised statement identified several goals at different timeframes. In the short term, achieving symptomatic remission and normalising C-reactive protein levels were prioritised. In the intermediate term, reducing calprotectin levels was identified as a target. Lastly, in the long term, the goals included achieving endoscopic healing and standardising the quality of life for individuals with IBD (Im et al., 2018; Turner et al., 2021). The subsequent therapies listed are commonly employed conventional treatments for ulcerative colitis.

### 3.1 Aminosalicylates

Aminosalicylates, alternatively referred to as 5-aminosalicylic acid (5-ASA) drugs, encompass a class of pharmaceuticals that possess the capability to mitigate inflammation within the gastrointestinal tract. These therapeutic agents function by specifically targeting the mucosal lining of the colon and rectum, which is the site of inflammation. Aminosalicylates are offered in diverse formulations, encompassing oral pharmaceuticals, rectal suppositories, and enemas. Corticosteroids are frequently employed as an initial therapeutic approach for individuals with mild to moderate ulcerative colitis, effectively managing symptoms and sustaining periods of remission. The class of medications known as 5-ASA-based drugs includes sulfasalazine, olsalazine, Mesalazine and balsalazide. These medications are widely recognised for their efficacy, safety, and affordability in the treatment of IBD, particularly UC (Sood et al., 2019).

### 3.2 Corticosteroids

Corticosteroids, such as prednisone and budesonide, exhibit potent anti-inflammatory properties and have demonstrated efficacy in promptly alleviating symptoms associated with moderate to severe ulcerative colitis exacerbations. These medications function through the inhibition of the immune system and the mitigation of inflammation within the colon. Corticosteroids are commonly prescribed for a limited duration to induce remission or manage acute symptoms. The avoidance of prolonged corticosteroid use is typically recommended due to the potential for significant adverse effects. According to Wild et al. (2003) and Waljee et al. (2016), these interventions effectively decrease intestinal inflammation by promptly reducing intestinal permeability, suppressing the production of Tumour necrosis factor (TNF) and inhibiting Nuclear factor-κB (NF-κB).

### 3.3 Immunomodulators

Immunomodulators represent a category of pharmaceutical agents that exert a modulatory or regulatory effect on the immune system. Patients who exhibit poor responses to aminosalicylates or corticosteroids are frequently prescribed these medications. Additionally, they may be used as a maintenance therapy to mitigate the occurrence of flare-ups., mercaptopurine (MP), Azathioprine (AZT) and methotrexate (MTX) are among the immunomodulators commonly employed in the treatment of ulcerative colitis. These pharmaceutical agents function by inhibiting the immune response and diminishing inflammation within the colon. According to Singh et al. (2022), in cases where patients with IBD do not respond to 5-ASA drugs and are reliant on or unresponsive to corticosteroids, it is advised to utilise conventional immunomodulators such as 6-MP, AZT, and MTX to maintain remission.

### 3.4 Biologic therapies

Biologic therapies are a relatively new class of pharmaceutical interventions that specifically target specific molecules or cells involved in the inflammatory pathway of ulcerative colitis. These interventions are usually recommended for people with moderate-to-severe symptoms who have not responded to conventional treatment. Biological therapeutics, such as anti-tumour necrosis factor (TNF) agents (e.g., adalimumab & infliximab) or integrin receptor antagonists (e.g., vedolizumab), function by inhibiting specific proteins or cells involved in the inflammatory processes taking place in the gastrointestinal tract. In contemporary medical practice, infliximab and adalimumab are the prevailing anti-tumour necrosis factor-alpha (TNF- α) agents that are frequently employed. The administration of these medications occurs through intravenous and subcutaneous routes, respectively. According to Seo and Chae (2014), clinical trials have provided evidence supporting the efficacy of infliximab and adalimumab in the treatment of moderate-to-severe UC.

### 3.5 Surgical interventions

Surgical interventions are necessary for treating ulcerative colitis when severe symptoms arise, complications arise, or conventional treatments are ineffective. Proctocolectomy involves removing the colon and rectum, with or without an ostomy. This procedure has high efficacy in eradicating the afflicted colon, providing patients with a prolonged period of remission. Treatment selection depends on the severity and scope of ulcerative colitis, as well as individual patient factors and preferences. (Kuhn and Klar, 2015).

## 4 Antibiotics used in UC treatment

Rifaximin modifies gut microbiota for small intestinal bacterial overgrowth and hepatic encephalopathy, while antibiotics aim to manipulate intestinal flora, potentially disrupting IBD progression. In their study, Khan et al. (2011) conducted a meta-analysis that examined the findings of 10 randomised controlled trials (RCTs) focused on the utilisation of antibiotics in patients diagnosed with active CD. Additionally, the study also included nine RCTs that investigated the impact of antibiotic usage in individuals with UC. Antibiotics significantly impact remission and disease severity in UC and CD. Rifamycin derivatives are effective in treating active CD. However, results vary due to different trials involving rifaximin, fluoroquinolones, macrolides, and anti-tuberculosis antibiotics. (Steinhart et al., 2002).

## 5 The role of gut microbiota in UC

The gut microbiota, an important group of microorganisms that live in the digestive tract, is correlated to several diseases, including ulcerative colitis. The composition and function of the microbiota in the gut have both been shown to be susceptible to change, as recent research has shown. The gastrointestinal tract of a neonate does not initially contain any microorganisms but is eventually colonised by bacteria that come from the mother and the environment. (Dominguez-Bello et al., 2010). According to Vyas and Ranganathan (2012), the number of bacterial cells present on and within the entire body of an adult human is approximately ten times greater than the total number of human cells. The complexity and diversity of the human microbiome are considerable. The composition and quantity of anatomical features vary across different regions of the gastrointestinal tract, ranging from the nasal and oral cavities to the distal segments of the colon and rectum. The composition and complexity of the gut microbiota exhibit variability during the transition of infants from a liquid diet to solid foods. The alterations in dietary patterns during adulthood play a substantial role in shaping the composition of the gut microbiota. Metagenomic techniques based on 16S ribosomal RNA sequence have improved our understanding of microbial communities in the gastrointestinal tract. This approach reveals that 90% of the bacterial population is attributed to Bacteroidetes and Firmicutes. (Mariat et al., 2009; Hu and Gubatan, 2023).

### 5.1 Dysbiosis in UC

Dysbiosis in the gut microbiota is the root cause of ulcerative colitis. This condition is characterised by a decrease in commensal bacteria such as *bifidobacteria* and *Lactobacilli* and an increase in pathogenic bacteria such as *Escherichia coli* and *Fusobacterium*. The imbalance that can result from dysbiosis can put a person at risk for several different diseases, including IBD and irritable bowel syndrome. Additionally, it has the potential to bring about unanticipated outcomes, such as the activation of an HIV infection or the development of autoimmune diseases. (Liu et al., 2016).

### 5.2 Impaired gut barrier function

The gut microbiota and intestinal epithelial cells collaborate to establish a defensive barrier aimed at impeding the infiltration of pathogens and toxins into the bloodstream. The integrity of the barrier function in ulcerative colitis is compromised, resulting in heightened permeability of the intestinal lining. The presence of dysbiosis in individuals with ulcerative colitis has been observed to have a detrimental effect on the integrity of the gut barrier. This compromised barrier allows for the infiltration of pathogenic bacteria and their associated metabolic by-products into the intestinal mucosa, subsequently eliciting an immune response. This phenomenon gives rise to atypical immune-inflammatory responses, including inflammation, allergies, and autoimmune disorders, which are facilitated by molecular mimicry and dysregulated T-cell reactions. According to the study conducted by Barnaba and Sinigaglia, 1997, it was found that the maintenance of intestinal homeostasis and the differentiation between harmful pathogens and beneficial commensal microbes heavily rely on the Treg/TH17 ratio, which represents the equilibrium between regulatory T cells (Tregs) and T helper type 17 cells (TH17). This regulation is strongly linked to the gut microbiota, as commensal microorganisms, including *Firmicutes, Bacteroides fragilis and Bifidobacterium infantis* promote the expansion of Treg cells, specifically FOXP3-expressing Treg and IL-10-producing Treg lymphocytes. Foxp3+ regulatory T cells play a crucial role in gut immunity and physiology, inducing intestinal tolerance and defending the host against harmful dietary antigens, commensal microorganisms, and pathogens. They also facilitate local tissue repair and maintain the integrity of the epithelial barrier, making them essential non-immune cells in the gastrointestinal tract. (Cosovanu and Neumann, 2020).

### 5.3 Immune system dysregulation

The gut microbiota exerts a substantial impact on the maturation and modulation of the immune system. Individuals diagnosed with UC exhibit an atypical immune response towards the gut microbiota, which is distinguished by an intensified activation of the immune system and the persistence of inflammatory processes. The dysregulation of the immune system can sustain the ongoing cycle of inflammation and subsequent tissue damage in the colon, thereby playing a role in the advancement of ulcerative colitis. The dysregulation of the immune response in the intestinal region is a crucial factor in the development of IBD, involving various molecules such as cytokines. In a recent study conducted by Kmiec et al. (2017), it was demonstrated that there is a correlation between the innate immune response and the promotion of gut inflammation in patients with IBD. The mucosa of patients with UC exhibits an altered equilibrium between regulatory T-cells and effector T-cells, including Th1, Th2, and Th17 cells. Evidence suggests that there is a correlation between UC and an a typical type 2 immune response, which is mediated by non-classical natural killer T-cells that produce IL-5 and IL-13. Previous studies have demonstrated that IL-13, which is secreted by specific subsets of NKT cells, plays a crucial role in exerting cytotoxic effects on epithelial cells. These effects include the induction of apoptosis and alterations in the composition of tight junction proteins (Fuss and Strober, 2008; Heller et al., 2008). Poggi et al. (2019) demonstrated a correlation between the exacerbation of pathology and the presence of supplementary inflammatory cytokines, including TNF, IL-1, IL-6, and IL-9.

### 5.4 Role of short-chain fatty acids

Short-chain fatty acids (SCFAs) are one of many metabolites produced by the gut microbiota during the fermentation of dietary fibres,exhibit anti-inflammatory properties. These fatty acids, typically not exceeding six carbon atoms, are derived from prebiotic or microbially fermentable carbohydrates like inulin, polysaccharides, and resistant starch. (Dalile et al., 2019) Butyrate, acetate, and propionate are examples of SCFAs that play important roles in intestinal health.The observed effects include acceleration of the regeneration and healing process of intestinal epithelial cells, augmentation of mucus production, and maintenance of appropriate pH levels within the intestine. Moreover, they hinder the attachment of pathogenic microorganisms to enterocytes. Acetate is commonly utilised as a cellular energy substrate for the growth and maintenance of muscle tissue and colonic cells. Butyrate exhibits a variety of beneficial effects on the host, such as strengthening metabolism, modulating the host’s immune system, and facilitating anti-inflammatory mechanisms. As a result, it attracts considerable attention. (Guarino et al., 2020) One of the hallmarks of UC is a decrease in SCFAs production, which contributes to an already severe inflammatory response and jeopardises the GI tract’s overall health. Because of this decline in SCFAs producers, it is common to find lower levels of SCFAs in faeces taken from people with inflammatory IBD. In a study of people with UC, Machiels et al. (2013) found lower levels of acetate and propionate in their faeces but not butyrate. When studying faecal samples from people with IBD Huda-Faujan et al. (2010) found lower levels of butyrate and propionate. Recent research findings indicate notable disparities in gut microbial species, microbial diversity and metabolic pathways between individuals diagnosed with IBD and healthy individual. (Lavelle and Sokol, 2020)

### 5.5 Potential therapeutic strategies

Due to the significant impact of the gut microbiota on ulcerative colitis, there is a growing interest in the development of effective strategies aimed at modulating the microbiota to restore equilibrium and improve disease outcomes. This involves the utilisation of probiotics, which are live beneficial bacteria that can be employed to reinstate the equilibrium of microorganisms in the gastrointestinal tract. Prebiotics, classified as dietary fibres that possess the ability to selectively enhance the proliferation of advantageous bacteria, can additionally be employed to enhance the growth of beneficial microorganisms. In addition, the utilisation of synbiotics, a combination of probiotics and prebiotics, may exhibit synergistic outcomes in the restoration of equilibrium within the gut microbiota (Hu and Gubatan, 2023). The present review provides an overview of the existing microbiome-centred therapeutic strategies employed in the management of IBD, as outlined in **Table 1**.

**Table 1:** Summary of current microbiome-based therapeutic strategies in IBD.

Therapeutic	Examples	Effects on microbiome
**Antibiotics**	Rifaximin Ciprofloxacin Metronidazole Tobramycin Amoxicillin	➢ Eliminate specific microbial populations that contribute to hyperinflammatory states to modulate IBD severity/disease activity ➢ Prevent overgrowth of harmful microbial species that may lead to secondary IBD complications (e.g. pouchitis and abscesses) ➢ Influence the development of anti-drug antibodies that affect the risk of immunogenicity to anti-TNF biologics ➢ Increase risk of microbial resistance ➢ Increase risk of infections (e.g. *Clostridium difficile*)
**Prebiotics (molecular compounds)**	Lactulose Psyllium Fructo-oligosaccharides Germinated barley foodstuff	Metabolised by gut microorganisms to form small molecule by-products (e.g. butyrate and short-chain fatty acids) that influence the local microenvironment to preferentially favour growth of certain flora
**Probiotics (living microorganisms)**	*Lactobacillus* spp. *Bifidobacterium* spp. *Saccharomyces* spp. Non-pathogenic *Escherichia coli*	➢ Strengthen intestinal barrier function by inhibiting apoptosis of intestinal cells ➢ Regulate immunity through genetic pathways (NF-κB, IL-6, TNF-α) or direct influence on T-cells ➢ Produce small molecules (lactic acid, hydroperoxides) that directly influence the growth patterns of other microbial strains Provide local survival competition for scarce resources with other microorganisms
**Synbiotics**	Combination of prebiotics and probiotics	➢ Optimise a combination of prebiotics and probiotics for maximum synergistic effect ➢ Prebiotic(s) are specifically chosen to select for the growth and survival of probiotic organism(s)

## 6 Probiotics: restoring balance in the gut

Probiotics can be traced back to ancient civilisations when humans first began eating fermented foods as part of their regular diet. It was hypothesised by Elie Metchnikoff that the beneficial bacteria found in yoghurt could improve the health of the gut microbiome. Mackowiak (2013) presented the concept of probiotics for the first time. These microorganisms can positively modulate the composition of the gut microflora and reduce the presence of pathogenic bacteria that release harmful compounds within the human gastrointestinal tract. In previous studies, a range of microorganisms have been employed for disease management, thereby giving rise to the term “probiotics”. Probiotics, derived from Greek, refer to living, non-pathogenic organisms with beneficial effects on hosts. Vergin, in 1954, coined the term “probiotic” to compare the harmful effects of antibiotics on intestinal microbiota with the beneficial effects of some beneficial bacteria. The term “probiotic” was subsequently redefined by Lilly and Stillwell as a biologically active product produced by a microorganism that enhances the growth of another microorganism. As a result, the term was subsequently defined by Fuller (1989) as non-pathogenic microorganisms that, when ingested, have a beneficial impact on the health or physiology of the host. The most recent definition proposed by the FDA and WHO in collaboration is that of a live microorganism that, when administered in adequate quantities, confers a health advantage to the host. Several probiotic microorganisms are commonly utilised, species of *Bifidobacterium* and *Lactobacillus*, have been reported to be the predominant and subdominant groups among gut microbes, respectively (Casellas et al., 2007; Markowiak and Śliżewska, 2017). Bacterial organisms capable of forming spores, primarily belonging to the genus *Bacillus*, are prevalent in this context (Derikx et al., 2016; Jin et al., 2023).

**Figure 1:**
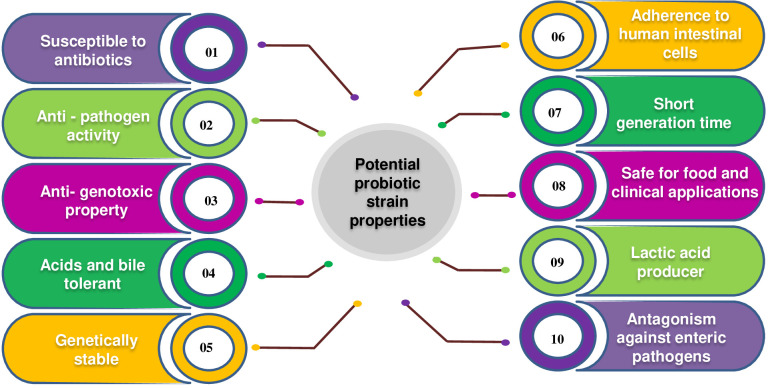
Characteristics of an ideal probiotic strain.

Probiotics are incorporated into various food products, particularly fermented dairy products, either individually or in conjunction with other strains. Continuously, novel genera and strains of probiotics are being discovered through increasingly sophisticated and targeted research endeavours. VSL#3 is a combination of eight distinct probiotic strains, exhibiting high strain specificity and variability in benefits across different patient groups. Probiotic products can consist of a solitary strain or a mixture of two or more strains, with the effectiveness of multi-strain probiotics demonstrated in controlled studies. (Chapman et al., 2011). The study of probiotics, particularly Lactobacilli, has experienced significant growth over the past twenty years. This is evident from the substantial increase in research articles published on the topic. Specifically, while there were approximately 180 research articles available between 1980 and 2000, the number of articles on the probiotic *Lactobacillus* exceeded 5700 between 2000 and 2014. According to a study published in PubMed in 2014, the effectiveness of the probiotic strain *Lactobacillus* was investigated. The characteristics of an ideal probiotic strain are depicted in **Figure 1**.

Probiotics act against intestinal diseases through various processes, including colonisation, which enhances their efficacy. They produce inhibitory constituents like organic acids, fatty acids, hydrogen peroxide, SCFAs, and bacteriocin-like inhibitory substances that prevent pathogens. (Tharmaraj and Shah, 2009). Bacteriocins refer to antimicrobial peptides or proteins that are produced by bacteria via ribosomal mechanisms. The aforementioned compounds demonstrate a diverse spectrum of antimicrobial efficacy against pathogenic bacteria, encompassing multiple strains of *Staphylococcus, Listeria, Bacillus, Clostridium*, and other bacterial species (Dai et al., 2021; Fathizadeh et al., 2022). The significance of bacteriocins synthesised by probiotic bacteria is ascribed to their secure utilisation in diverse domains, including the food industry, pharmaceuticals, veterinary medicine, and human healthcare (Barcenilla et al., 2021; Sharma et al., 2021; Somashekaraiah et al., 2021). The compounds cited in the scholarly articles (Sheoran and Tiwari, 2019; Sheoran and Tiwari, 2020; Chen et al., 2021) exhibit inhibitory effects on a wide range of bacteria. Their mechanism of action involves reducing cell viability, modifying bacterial cell metabolism, and inhibiting toxin production. Additional mechanisms of probiotic action include competitive inhibition on the surface of the intestinal epithelium, wherein the probiotics obstruct the binding sites of the intestinal epithelial surface, as well as a reduction in the interaction between the pathogen and the host. Similarly, the investigation of nutrient competition is also examined as a mechanism underlying probiotic activity. According to the suggestions of the World Health Organization (WHO), Food and Agriculture Organization (FAO), and the European Food Safety Authority (EFSA), in their selection process, probiotic strains must meet both safety and functionality criteria, as well as those related to their technological usefulness. Probiotic characteristics are not associated with the genus or species of a microorganism, but with few and specially selected strains of a particular species (Hill et al., 2014). FAO and WHO have jointly developed guidelines that propose a systematic approach for conducting comprehensive evaluations of probiotics in food products, to provide scientific evidence to support health claims and benefits. The essential criteria for an ideal probiotic are presented by Pandey et al. (2015). The application of the FAO/WHO guidelines on Probiotics has the potential to serve as a universal benchmark for evaluating probiotics in food products, thereby facilitating the validation of health-related assertions.

The guidelines stipulate that the following activities must be carried out:

The identification and categorisation of strains.

• A comprehensive elucidation of the strain(s) about their safety and probiotic attributes.
• Verification of the health benefits observed in human studies.
• Ensuring the accuracy and transparency of efficacy claims and product information throughout the entire duration of the product’s shelf life.

### 6.1 Probiotics in ulcerative colitis

The colon contains the highest concentration of microorganisms, suggesting treating colon microbiome abnormalities could benefit individuals with ulcerative colitis. Studies have shown potential benefits from various probiotic strains. (Basso et al., 2019). Previous studies have demonstrated that the use of the non-pathogenic strain of E. coli Nissle 1917 (EcN) exhibited similar efficacy and safety characteristics in the maintenance therapy of patients with mild or moderate ulcerative colitis when compared to treatment with salicylates (Basso et al., 2019; Bischoff et al., 2020). The potential therapeutic and prophylactic effects of the probiotic strain EcN against adherent-invasive Escherichia coli (AIEC) infection in zebrafish were investigated. In the zebrafish model, the researchers discovered that EcN effectively reduced AIEC colonisation, tissue damage, and pro-inflammatory responses. Furthermore, EcN reduced AIEC hyperinfection in zebrafish, especially when propionic acid was present. The effectiveness of EcN in combating AIEC infection in a zebrafish model is highlighted in this study. (Nag et al., 2022) The utilisation of various strains of lactic acid bacteria and *bifidobacteria* as supplementary treatment has been observed to significantly improve the progression of the disease and the sustained absence of symptoms in individuals diagnosed with ulcerative colitis (Basso et al., 2019). A highly regarded probiotic complex is VSL#3, which consists of four strains of *Lactobacillus (L. casei, L. acidophilus, L. plantarum* and *L. delbrueckii* subsp. *bulgaricus*), three strains of *Bifidobacterium (B. longum, B. breve*, and *B. infantis)*, and one strain of *Streptococcus* (*S. salivarius* subsp*. termophilus*) (Basso et al., 2019). Studies conducted on mouse models have demonstrated that the administration of this probiotic mixture leads to the suppression of NF-B and TNF expression in the TLR4-NF-B signalling pathway. Consequently, the downregulation of pro-inflammatory cytokines and toll-like receptors (TLR) occurs while the upregulation of regulatory cytokines is observed (Jakubczyk et al., 2020; Silva et al., 2020). Previous studies have demonstrated the efficacy of VSL#3 in inducing and sustaining remission in individuals with mild to moderate UC when used as adjuvant therapy or as a standalone treatment (Basso et al., 2019).

In a systematic review and meta-analysis conducted by Derwa et al. (2017), it was found that the VSL#3 probiotic mixture demonstrated potential efficacy in inducing remission among individuals with UC. Furthermore, the study suggested that the use of VSL#3 probiotics may be comparable to 5-5-ASA in terms of preventing disease exacerbations. Under the recommendations put forth by the European Society for Clinical Nutrition and Metabolism (ESPEN), the investigation of probiotics such as VSL#3 and EcN in the treatment of mild to moderate UC is warranted, as they have shown potential for inducing remission in affected patients. According to Bischoff et al. (2020), the use of probiotics is contraindicated in cases of severe ulcerative UC. The use of probiotics as a supplementary treatment may have particular efficacy in the management of patients who experience intolerance to 5-ASA (Miele et al., 2018; Bischoff et al., 2020). **Table 2** presents several clinical trial findings regarding the use of probiotics for the treatment of UC.

**Table 2:** Some clinical trial data of probiotics for treating UC.

Sr. no.	Probiotic Used	Outcome	Reference
1	A probiotic product that contained *L*. *casei* Zhang*, L*. *plantarum* P*-*8 and *B. animalis* subsp*. lactis*	The overall remission rate was 91.67% for the probiotic group vs. 69.23% for the placebo group (*P* = 0.034)	Chen et al., 2020a.
2	Symprove (contains *Lactobacillus rhamnosus* NCIMB 30174, *Lactobacillus plantarum* NCIMB 30173, *Lactobacillus acidophilus* NCIMB 30175 and *Enterococcus faecium* NCIMB 30176	The calprotectin levels were significantly decreased following 4 weeks in the probiotic group (p = 0.011 and 0.001, t-test and Wilcoxon’s, respectively)	Bjarnason et al., 2019.
3	Kefir (*Lactobacillus* Bacteria)	No statistically significant difference was found between weeks 1 and 2 in patients with UC in terms of abdominal pain, bloating, frequency of stools, defecation consistency, and feeling good.	Yilmaz et al., 2019.
4	A tablet contains *Streptococcus faecalis* T-110, *Clostridium butyricum* TO-A and *Bacillus mesentericus* TO-A	At 12 months, the remission rate was 69.5% in the treatment group and 56.6% in the placebo group (p = 0.248). The relapse rates in the treatment and placebo groups were 0.0% vs. 17.4% at months (p = 0.036).	Yoshimatsu et al., 2015.
5	Mil–Mil (a fermented milk product containing B. *breve* strain Yakult and *Lactobacillus acidophilus*	Relapse-free survival was not significantly different between the treatment and placebo groups (*P* = 0.643)	Matsuoka et al., 2018.
6	*Escherichia coli* Nissle 1917	Efficacy in maintaining remission and preventing relapse comparable to Mesalazine	Kruis et al., 2004.
7	*Escherichia coli* Nissle 1917	Efficacy in maintaining remission after exacerbation of UC comparable to mesalazine	Rembacken et al., 1999.
8	*Escherichia coli* Nissle 1917	Efficacy and safety in maintaining remission comparable to mesalazine	Kruis et al., 2004.
9	*Escherichia coli* Nissle 1917	Efficacy in maintaining remission comparable to mesalazine	Henker et al., 2008.
10	*Escherichia coli* Nissle 1917	Possibility of dose-dependent efficacy in inducing remission of the rectal probiotic compared to placebo	Matthes et al., 2010.
11	*Escherichia coli* Nissle 1917	No benefit in the use of probiotics as an additional therapy to conventional treatment	Petersen et al., 2014.
12	*Lactobacillus* GG	Higher efficacy of probiotics as add-on therapy in prolonging the relapse-free time compared to mesalazine monotherapy	Zocco et al., 2006.
13	*Bifidobacterium breve, Bifidobacterium bifidum, Lactobacillus acidophilus* YIT 0168 (*Bifidobacteria*-Fermented Milk- BFM)	Higher efficacy of probiotic mixture as add-on therapy in maintaining remission and preventing relapse compared to conventional therapy alone	Ishikawa et al., 2003.
14	*Bifidobacterium breve, Bifidobacterium bifidum, Lactobacillus acidophilus* YIT 0168 (*Bifidobacteria-Fermented* Milk- BFM)	Higher efficacy of probiotics as add-on therapy in maintaining remission compared to conventional therapy alone	Kato et al., 2004.
16	*Lactobacillus reuteri* ATCC 55730	Higher efficacy of probiotic enema as add-on therapy additional to oral mesalazine in improving mucosal inflammation compared to conventional therapy	Oliva et al., 2011.
17	*Lactobacillus casei, Lactobacillus plantarum, Lactobacillus acidophilus and Lactobacillus delbrueckii* subsp. *Bulgaricus, Bifidobacterium longum, Bifidobacterium breve* and *Bifidobacterium infantis, Streptococcus salivarius* subsp. *Thermophils* (VSL#3)	The higher efficacy of probiotic mixture as add-on therapy to conventional treatment in patients with the relapsing disease compared to placebo	Tursi et al., 2010.
18	*Lactobacillus casei, Lactobacillus plantarum, Lactobacillus acidophilus and Lactobacillus delbrueckii* subsp. *Bulgaricus, Bifidobacterium longum, Bifidobacterium breve* and *Bifidobacterium infantis, Streptococcus salivarius* subsp. *Thermophils* (VSL#3)	Higher efficacy in inducing and maintaining remission compared to placebo	Sood et al., 2009.
19	*Lactobacillus casei, Lactobacillus plantarum, Lactobacillus acidophilus* and *Lactobacillus delbrueckii* subsp*. Bulgaricus, Bifidobacterium longum, Bifidobacterium breve* and *Bifidobacterium infantis, Streptococcus salivarius* subsp*. Thermophils* (VSL#3)	Higher efficacy in maintaining remission compared to placebo	Miele et al., 2009.
20	*Lactobacillus plantarum*, *Bifidobacterium longum*, and *Bifidobacterium bifidum*	Suppressed colonic inflammation, and fatigue by the suppression of the IL-1β or IL-6 to IL-10 expression ratio and gut bacterial LPS production	Yoo et al., 2022.
21	*Lactobacillus plantarum*	Restored gut microbiota balance and modulated the resident gut microbiota and immune response	Khan et al., 2022.
22	*Ligilactobacillus salivarius* Li01 and RSV	An improved synergistic anti-inflammatory effect from the RSV and Li01 combination treatment	Fei et al., 2022.
23	Goji juice fermented by *Lactobacillus plantarum, Lactobacillus reuteri* and *Streptococcus thermophilus*	Probiotics-fermentation enhanced the anti-ulcerative colitis function of goji berry juice and modulated gut microbiota	Liu et al., 2021.
24	*Lactobacillus plantarum* CBT LP3 (KCTC 10782BP)	Effective anti-inflammatory effects, with increased induction of Treg and restoration of goblet cells, suppression of proinflammatory cytokines	Kim et al., 2020.
25	*Bifidobacterium breve*, CCFM683	Improved intestinal epithelial barriers, restored gut microbiota	Chen et al., 2020b.
26	*Bifidobacterium infantis, Lactobacillus acidophilus, Enterococcus faecalis* with (quadruple probiotics, Pqua) or without (triple probiotics, P-tri) aerobic *Bacillus cereus*	Effective (Aerobe-contained Piqua was a powerful adjuvant therapy for chronic colitis, via restoring the intestinal microflora and recovering the multi-barriers in the inflamed gut)	Chen et al., 2020c.
27	*Lactococcus lactis* subsp. lactic JCM5805	Effective (high-dose administration deteriorates intestinal inflammation)	Komaki et al., 2020.
28	*Lactobacillus bulgaricus*	Regulates the inflammatory response and prevents Colitis-associated cancer	Silveira et al., 2020.
29	*Lactobacillus* and *Bifidobacterium* species	Significantly induced remission in UC patients	Agraib et al., 2022.
30	*Bacillus coagulans* Unique IS-2	Showed beneficial effects when administered along with standard medical treatment	Bamola et al., 2022.
31.	*Bifid Triple Viable Capsules*	IL-1, TNF-α, and IL-10 had higher decreases in a test group	Cui et al., 2004.
32	*Lactobacillus casei* DG	Both orally and rectally given probiotics have shown SS improvement in clinical and histological scores	D’Inca et al., 2011.
33	*Bifidobacterium longum*	Sigmoidoscopy scores (SS) and blood-serological markers TNF- α and IL-1 were reduced. Both clinical activity index (CAI) and bowel habit index (BHI) were reduced in a test group	Furrie et al., 2005.
34	*Bifidobacterium breve*	The endoscopic score of the treatment group was significantly lower. Myeloperoxidase analysis (MPO) amounts in the lavage solution (LS) significantly decreased	Ishikawa et al., 2011.
35	*Bifid Triple Viable Capsules*	Higher decrease in UCDAI scores and symptoms in the test group. TNF-α and IL-8 were decreased in a test group	Huang et al., 2018.
36	*Enterococcus faecium, Lactobacillus plantarum, Streptococcus thermophilus, Bifidobacterium lactis, Lactobacillus acidophilus, Bifidobacterium longum*	SS differences in decrease of endoscopic and clinical index score. The test group achieved a higher decrease	Kamarli et al., 2019.
37	*Bifidobacterium breve, Bifidobacterium bifidum, Lactobacillus acidophilus* YIT 0168	CAI score, endoscopic score, and histological score were significantly lower in the treatment group	Kato et al., 2004.
38	*E.coli* Nissle 1917 (Serotype O6: K5: H1)	No significant differences both in CAI scores and relapse rates. Relapse-free time differences were also NS	Kruis et al., 1997.
39	*E.coli* Nissle 1917 (Serotype O6: K5: H1)	NS differences in decrease of clinical symptoms and blood-serological markers between groups. Both groups had decreased inflammation markers and symptoms	Arribas et al., 2009.
40	*Bifidobacterium breve, Bifidobacterium bifidum, Lactobacillus acidophilus* YIT 0168	NS differences in both relapse-free survival and clinical deterioration	Matsuoka et al., 2018.
41	*E.coli* Nissle 1917 (Serotype O6: K5: H1)	The dose depended on efficacy in both remission time and endoscopic findings	Matthes et al., 2010.
42	*L. paracasei, L. plantarum, L. acidophilus, L. delbrueckii subsp bulgaricus, B. longum, B. breve, B. infantis, Streptococcus thermophilus*	More patients achieved remission in the test group	Ng et al., 2010.
43	*Lactobacillus salivarius, Lactobacillus acidophilus, Bifidobacterium bifidus strain BGN4*	The better improvement compared to the control	Palumbo et al., 2016.
44	*Bifidobacterium longum BB536*	Significant decrease in UCDAI scores and endoscopic index in a test group	Tamaki et al., 2015.
45	*L. acidophilus* strain LA-5 and *B. animalis* subsp. *lactis* strain BB-12	More patients in the test group achieved remission. Median relapse time was longer in a test group	Wildt et al., 2011.
46	*Bifid Triple Viable Capsules*	The observation group had significantly lower scores in CDAI and UCAI as well as recurrence rate	Fan et al., 2019.
47	*Lactobacillus* spp.	Reduced fecal calprotectin (FCAL) in UC patients. No differences in IBD-QOL scores and blood-serological markers	Yilmaz et al., 2019.
48	*Bifidobacterium* and *Lactobacillus* with sulfasalazine and prednisone vs. sulfasalazine	Level decrease of CRP, TNF-α and IL-10 in both groups, significantly lower in the study group (p < 0.05); significantly higher treatment effect in the study group (p < 0.05); higher infection rate in the control group (p < 0.05)	Su et al., 2018.
49	VSL#3	75% of patients remained in remission during the study period, with no side effects	Venturi et al., 1999.
50	*Eschericia coli* Nissle 1917 (1×1011cfu/day)	Prebiotics induce remission as effectively as mesalazine (standard treatment)	Rembacken et al., 1999.
51	VSL#3	VSL#3 resulted in combined induction of remission/response rate of 70%, with no adverse effects	Bibiloni et al., 2005.
52	*Saccharomyces boulardii*	71% of patients remained in remission	Guslandi et al., 2003.
53	Yakult (1×1010 cfu/day)	73% of patients treated with probiotics remained in remission, while only 10% of placebo	Ishikawa et al., 2003.

## 7 Prebiotics: nourishing beneficial bacteria

The term “prebiotics” is a relatively recent concept that is clearly defined as “nondigestible food ingredients that beneficially alter the host by selectively stimulating the growth and/or activity of a specific group of bacteria in the colon, thereby improving the overall health of the host” (Gibson and Roberfroid, 1995). Recently, the International Scientific Association for Probiotics and Prebiotics (ISAPP) convened a panel of experts to reassess the definition of prebiotics. The panel has expanded the definition to encompass a broader understanding, defining it as “a substrate that is selectively utilised by host microorganisms, resulting in a health benefit.” The definition presented by Gibson et al. (2017) expands upon the concept of prebiotics by considering the potential inclusion of non-carbohydrate substances, exploring their relevance to body sites beyond the gastrointestinal tract, and identifying distinct groups beyond those found in food. These substances are typically classified as short-chain carbohydrates that are resistant to digestion yet serve as substrates for the proliferation of probiotic microorganisms within the upper gastrointestinal tract. Cocoa-derived flavanols are compounds that are not classified as carbohydrates but have been proposed as potential prebiotics. According to Tzounis et al. (2011), experimental studies conducted both *in vivo* and *in vitro* demonstrate that flavanols can enhance the proliferation of lactic acid bacteria.

**Figure 2:**
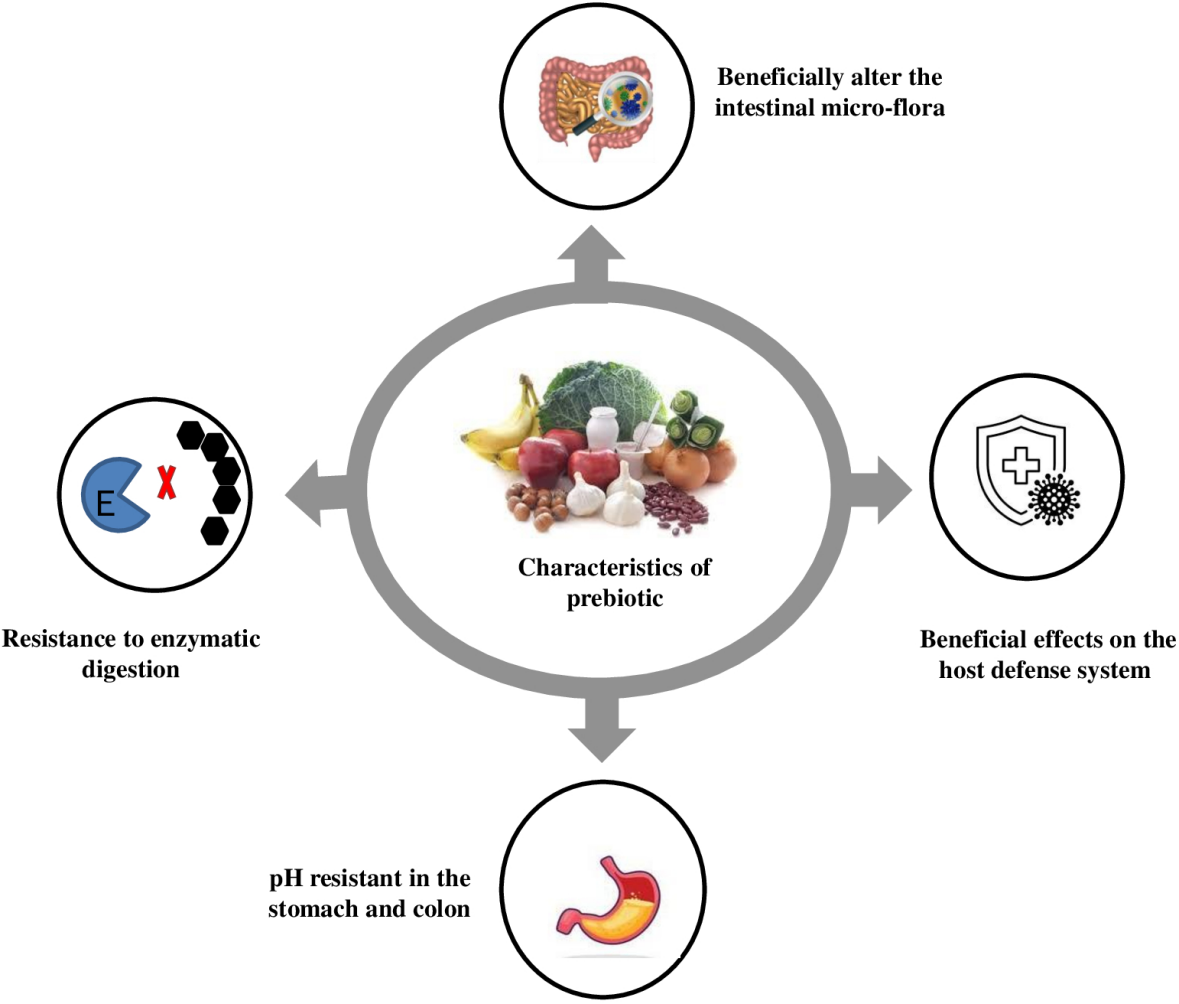
Characteristics of prebiotic.

A significant increase in the *Bifidobacteria* population in faecal samples has been reported due to fructooligosaccharides (FOS) consumption. Prebiotics, consisting of glucose, fructose, galactose and/or xylose, undergo minimal hydrolysis in the intestinal tract and have caloric value due to their resistance to digestion and energy metabolism through fermentation (Roberfroid, 1993). Prebiotics like FOS, isomalto-oligosaccharides (IMO), and xylooligosaccharides (XOS) are studied for their potential benefits on stool volume, constipation relief, and faecal acidity. FOS, like inulin and neosugar, are dietary fibres that improve stool volume and faecal acidity. These prebiotics are easily metabolised by *bifidobacteria* and other microorganisms like *Lactobacillus acidophilus, Bacteroides vulgatus, B. ovatus, B. thetaiotaomicron, B. fragilis*, and *Enterococcus faecium, E. faecalis* (Guarino et al., 2020) An optimal prebiotic substance should possess several key characteristics, as illustrated in **Figure 2**. IMO can be identified in various fermented food products, including miso, soy sauce, and honey. These compounds are known to be metabolised by *bifidobacteria* and the Bacteroides groups. IMO facilitates the proliferation of Bifidobacterium and *Lactobacillus* species, leading to both local and systemic Th1-like immune responses and the regulation of immune function. The presence of positive outcomes has been identified in the clinical trials. XOS are a class of prebiotics that occur naturally in various sources such as fruits, bamboo shoots, vegetables, milk, honey, and others (Aachary and Prapulla, 2010). Previous research has indicated that Bifidobacterium adolescentis can utilise XOS, while*, Lactobacillus rhamnosus*, *L. plantarum* and *Lactococcus lactis* have been found to efficiently metabolise oat -glucooligosaccharides. According to Van Laere et al. (2000), the fermentation process of arabinose-XOS derived from wheat meal by *bifidobacteria* can be attributed to the existence of xylanolytic enzyme systems such as xylosidase and a limited number of arabinosidases. The presence of a -D-xylosidase derived from *Bifidobacterium breve* K-110, as well as arabinosidases originating from *B. adolescentis* DSM20083 (Van Laere et al., 2000) and *B. breve* (Shin et al., 2000), has been documented in the literature. Zeng et al. (2007) conducted a study on the species *B. bifidum, B. adolescentis*, and *B. infantis*, and reported that these species exclusively exhibited arabinosidase and xylosidase activity, while no activity of, glucuronidase, or acetyl xylan esterase and xylanase was observed. Gullon et al. (2008) studied the fermentation process of XOS from rice husks using probiotic bacteria. They found that *Bifidobacterium adolescentis* CECT 5781 showed significantly greater growth compared to *Breve* CECT 4839, *Bifidobacterium longum* CECT 4503 and Infantis CECT 4551 in the presence of XOS. This highlights the importance of industrial development in prebiotics for enhancing food quality and human health.

A variety of functional foods containing prebiotics have been utilised in the production of biscuits, candies, tabletop sweeteners, frozen yoghurt, and other similar products (Davani-Davari et al., 2019). In Japan, the Foods for Specified Health Uses (FOSHU) programme has incorporated various prebiotics, such as FOS, IMO, XOS, lacto-sucrose, and lactulose oligosaccharides, into specific food products. Additional examples of prebiotic foods include yoghurts, cereals, cakes; cereal bars biscuits high in dietary fibre, powdered beverages, pasta, sauces, bread, infant formula products and various fruit juices (Desai, 2008). Prebiotics are commonly associated with specific types of dietary fibre, such as inulin and FOS. However, they can also be found in various food sources, including cultivated plants and wild plants. Below are several examples of food plants that are abundant in prebiotics; categorised accordingly as cultivated and wild plants.

Cultivated plants

• Bananas: Bananas contain FOS, which is a type of prebiotic that is fermented by beneficial bacteria in the gut. FOS has been shown to advance gut health, decrease inflammation, and improve the immune system. (Sabater-Molina et al., 2009)
• Garlic: Garlic contains inulin, a prebiotic fibre that nourishes gut bacteria. It is also known for its antimicrobial properties. (Davani-Davari et al., 2019)
• Legumes: Legumes, such as beans, lentils, and peas, are excellent sources of resistant starch, a type of prebiotic that is fermented by beneficial bacteria in the gut. Resistant starch has been shown to increase gut health, decrease the risk of chronic diseases, and benefit weight management. (Mirmiran, 2014)
• Oats: Oats are a great source of beta-glucans, a category of soluble fibre that is fermented by beneficial bacteria in the gut. Beta-glucans has been shown to improve gut health, reduce cholesterol levels, and help with weight management. (Sabater-Molina et al., 2009)
• Whole grains: Whole grains, such as brown rice, quinoa, and barley, are excellent sources of resistant starch and other prebiotics. (Sabater-Molina et al., 2009)
• Onion: Like garlic, onions are rich in inulin and can promote the growth of helpful gut bacteria. (Davani-Davari et al., 2019)
• *Asparagus*: *Asparagus* is a good source of inulin, a type of soluble fibre that is fermented by beneficial bacteria in the gut. Inulin has been shown to improve gut health, boost immunity, and decrease the risk of chronic disorders. (Sabater-Molina et al., 2009)
• *Jerusalem artichoke*: *Jerusalem artichokes* are high in inulin content, making them an excellent prebiotic food. (Sabater-Molina et al., 2009)
• Chicory Root: Chicory root is often used as a source of inulin in supplements, which is proven to increase the growth of *Bifidobacterium*. It can be consumed raw or roasted (Davani-Davari et al., 2019).

Wild Plants:

• *Taraxacum officinale* (Dandelion greens): A very common plant, dandelion grows wild almost everywhere. Dandelion is a native of Europe. In India, it is found in the Himalayas. Nutritionally, the dandelion has remarkable value. Dandelion greens are wild plant that contains inulin and other prebiotic fibres, along with various nutrients (Wirngo et al., 2016).
• *Arctium lappa L* (Burdock Root): Burdock root is a wild plant that contains inulin and has been traditionally used as a medicinal food with prebiotic properties. This species is native to the temperate regions of the Old World, from Scandinavia to the Mediterranean, and from the British Isles through Russia, and the Middle East to India, China, Taiwan and Japan. (Moro and Clerici, 2021).
• *Cichorium intybus* L (Chicory Greens): Wild chicory greens are rich in inulin and can be consumed in salads or cooked as a side dish. The Chicory crop is cultivated in a few States, mostly Uttar Pradesh, and Gujarat. These two states account for 97% of the total production of Chicory in India (Nwafor et al., 2017).
• *Musa paradisiaca* (Plantain): Plantain leaves are a common wild plant that contains prebiotic fibres, among other beneficial compounds. Plantain fruit is widely consumed in Nigeria, Africa and some other parts of the world (Ukwah et al., 2014).
• *Urtica Dioica* (Nettles): Nettles are known for their numerous health benefits and can provide prebiotic effects due to their fibre content. It is most common in Europe, North America, North Africa, and parts of Asia (Behzad et al., 2018).

These are just a few examples, and there are many other cultivated and wild plants that can offer prebiotic benefits. Combining a type of these prebiotic-rich foods into your diet can aid in promoting a healthy gut microbiome (Crittenden and Playne, 2008; Davani-Davari et al., 2019). For a product (food or supplement) to be believed to be a prebiotic, it must meet the below-mentioned conditions (Gibson et al., 2017).

Prebiotic enhance the proliferation and metabolic function of selected bacterial strains that exhibit beneficial impacts on overall well-being in the following ways.

• Lower the pH of the substances presents in the intestines.
• To exhibit resistance to hydrolysis and the effects of gastrointestinal enzymes.
• To avoid being absorbed in the upper gastrointestinal tract.
• To maintain a suitable environment for one or more beneficial microorganisms within the colon.
• To maintain stability throughout the food processing procedure,

### 7.1 Prebiotics in UC

**Figure 3:**
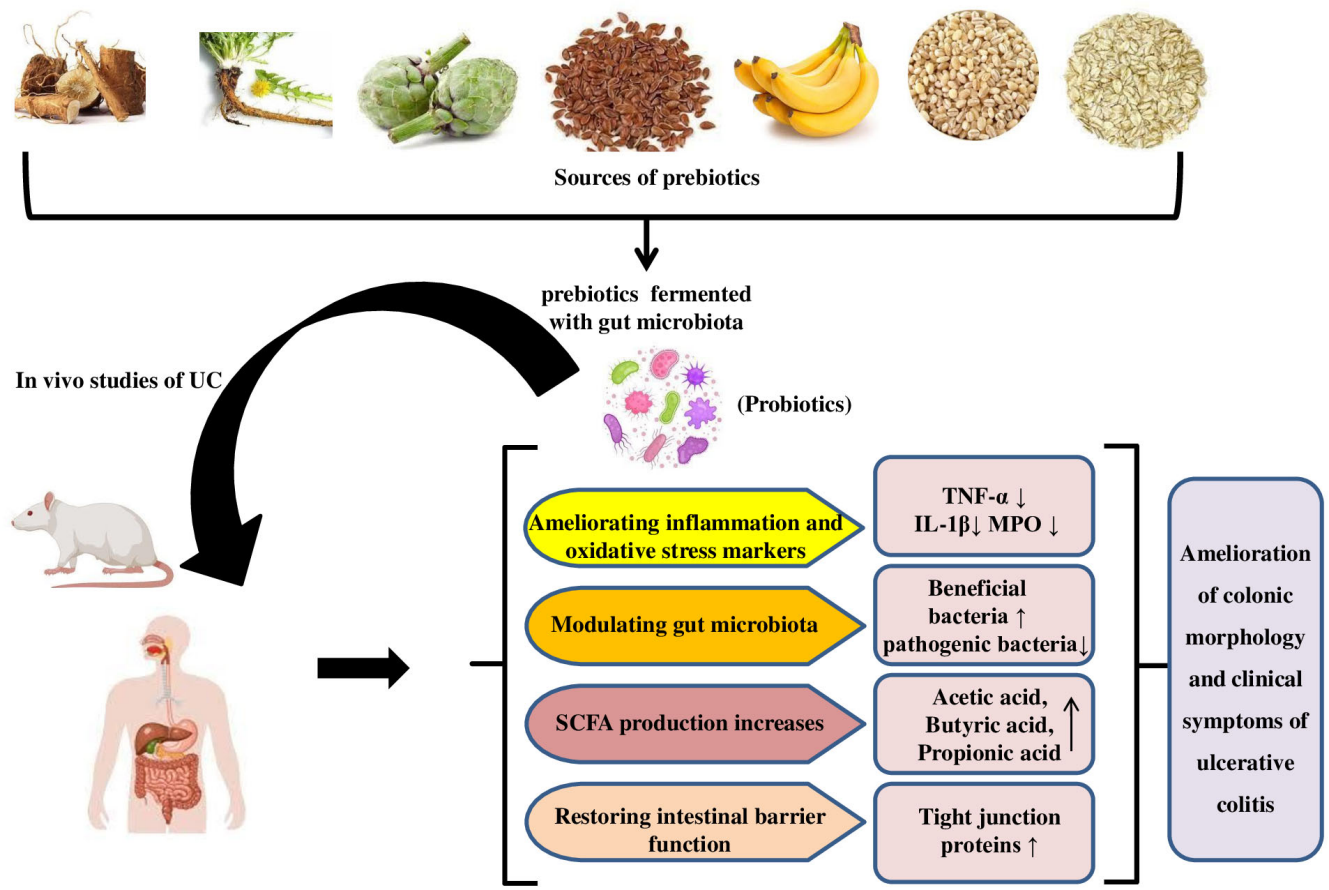
Mechanism for ameliorative effect of prebiotics in UC studies.

Prebiotics, mostly fermentable carbohydrates, promote local or systemic health (Pandey et al., 2015; Akram et al., 2019). Prebiotics can alter the intestinal microbiota, improve the intestinal barrier, and promote the growth of beneficial microbes in the digestive tract, which produce host-beneficial metabolites. (Aljuraiban et al., 2023) Like probiotics, clinical trials on prebiotics in specific diseases are difficult to prove. Thus, prebiotic data in UC patients is carefully regulated. Prebiotics may help treat UC by supplementing fermentable carbohydrate-containing fibre fractions. Promoting specific bacteria and/or their metabolites achieves this (Pandey et al., 2015; Rasmussen and Hamaker, 2017). Prebiotics may help maintain remission or low clinical disease activity. Prebiotics used in UC studies are oligosaccharides and inulin (Rasmussen and Hamaker, 2017). The mechanism for the ameliorative effect of prebiotics in UC studies is illustrated in **Figure 3**. Prebiotics encourage the growth of beneficial microorganisms, which compete with harmful species and produce beneficial fermentation substances like SCFAs, which have immunomodulatory properties and influence toll-like receptor-4 signalling and pro-inflammatory cytokines. (Van der Beek et al., 2017)

Casellas et al. (2007) studied the effects of mesalazine treatment, oligofructose-enriched inulin, and placebo on patients with mild to moderate UC. Oral oligofructose-enriched inulin was well-received and reduced faecal calprotectin levels. Faecal calprotectin has been employed as a reliable and quantifiable indicator of intestinal inflammation, with a significant correlation observed between its levels and the assessment of disease activity in UC, as determined through histologic and endoscopic methods (Konikoff and Denson, 2006).In Japan, germinated barley food (GBF) has been explored as a therapeutic intervention for UC. GBF, a dietary fibre and protein-rich in glutamine, has been shown to reduce clinical activity in individuals with UC and is an effective treatment option for maintaining remission. The safety profile of the treatment is noteworthy, as the study conducted by Hanai et al. (2004) found no incidence of side effects in the study group associated with the use of GBF. Nevertheless, further clinical trials are required to provide additional confirmation regarding the efficacy of dietary fibre as a prebiotic in the management of UC. Prebiotics has been extensively utilised in the management and treatment of UC. **Table 3** presents a comprehensive compilation of various studies that have examined the advantageous effects of prebiotics in the management of UC.

**Table 3:** Studies of prebiotics in the management of UC.

Sr. No.	Prebiotic used	Outcome Results	Reference
1	Plantago ovata seed	Maintain remission as effectively as mesalamine, Increased in fecal butyrate.	Fernandez-Banares, 1999.
2	Synergy 1 (inulin and oligofructose) 6 g/day and Bifidobacterium longum	Reduction of defensins 2, 3, and 4, TNF - α and IL- 1	Furrie et al., 2005.
3	Germinated barley foodstuff (30 g/day)	Reduction of clinical activity index scores and increase in stool butyrate concentrations.	Mitsuyama et al., 1998.
4	Polysaccharide from *Scutellaria baicalensis* Georgi	Effective (attenuated body weight loss, reduced DAI, ameliorated colonic pathological damage, and decreased MPO activity)	Cui et al., 2021.
5	Synthetic glycans	Synthetic glycans increase survival, reduce weight loss, and improve clinical scores in mouse models of colitis	Tolonen et al., 2022.
6	GOS, FOS along with FMT	Treatment with FMT plus a prebiotic blend restores the structure of the intestinal flora and increased the levels of acetic acid, butyric acid, FFAR3, and ZO-1	Qian et al., 2022.
7	Alpha D-glucan from marine fungus *Phoma herbarum* YS4108	Effective (significantly increased butyrate, isovaleric acid levels, and prominent alterations on specific microbiota)	Liu et al., 2016.
8	Stachyose	Increased beneficial microbiota and bacterial diversity to alleviate acute colitis in mice	He et al., 2020.
9	*Dictyophora indusiate* polysaccharide	Effective (modulates gut microbiota)	Kanwal et al., 2020.
10	FMG or dealcoholised muscadine wine	Effective (reduced dysbiosis in the colon)	Li et al., 2020.
11	GFO	Effective (prevented and attenuated colitis symptoms and GI dysmotility, reducing populations of harmful bacteria and increasing SCFAs)	K-da et al., 2020.
12	β-fructans (oligofructose and inulin)	Did not prevent symptomatic relapses in UC patients but reduced the severity of biochemical relapse and increased anti-inflammatory metabolites	Valcheva et al., 2022.
13	Oral microencapsulated sodium butyrate (BLM)	BLM supplementation appears to be a valid add-on therapy to maintain remission in patients with UC	Vernero et al., 2020.
14	Oligofructose-enriched inulin	Less significant (scFOS on rectal sensitivity may require higher doses and may depend on the subgroup)	Valcheva et al., 2018.

## 8 Synbiotics

Gibson’s proposition of prebiotics was accompanied by a rationale for the potential advancements that could be achieved through the combination of prebiotics with probiotics, leading to the establishment of what he referred to as Synbiotics (De Vrese and Schrezenmeir, 2008). The term “synbiotics” pertains to the amalgamation of prebiotics and probiotics to improve the well-being of humans or animals (Markowiak and Śliżewska, 2017). According to research conducted by Perrin et al. (2001) and Sharma and Shukla (2016), the probiotic bacteria found in synbiotic food products make selective use of prebiotics as a substrate for their growth. A group of specialists from the International Scientific Association for Probiotics and Prebiotics got together and rethought the concept of synbiotics. Synbiotic treatment aims to improve the endurance and metabolic function of beneficial probiotic strains in the gut microbiota. Common combinations include lactobacilli and *bifidobacteria* with oligosaccharides, inulin, or fibres as prebiotics. This approach reduces systemic inflammation by increasing the population of bacteria producing SCFAs and providing substrates for fermentation (Pandey et al., 2015).

According to their classification, synbiotics can be categorised into two distinct classes: complementary and synergistic. A complementary synbiotic is comprised of a probiotic and a prebiotic, which collectively provide one or more health benefits without requiring interdependent functions. According to Swanson et al. (2020), a synergistic synbiotic is composed of a substrate that is specifically utilised by microorganisms that are co-administered. These guidelines are expected to be efficacious in establishing expectations regarding the comprehension of the interplay between pre- and probiotics as well as the advancement of synbiotic products for the promotion of health and therapeutic interventions. Numerous reports suggest that the consumption of synbiotic foods has a beneficial impact on the health and nutritional status of the host. The study conducted by Yang et al. (2005) revealed that the administration of synbiotics had a dual effect on the faecal sample. Firstly, it was observed that synbiotics increased the abundance of probiotic bacteria, specifically *Lactobacillus* and *Bifidobacterium*, while simultaneously reducing the presence of coliform bacteria. Additionally, the test group exhibited an improvement in the levels of various digestive enzymes, including lactase, sucrase, lipase and isomaltase. Recently, there has been a report indicating that the consumption of synbiotics has significantly decreased various cardiovascular risk factors, the prevalence of metabolic syndrome, and markers of insulin resistance among elderly individuals (Cicero et al., 2021). The ability of *bifidobacteria* to metabolise prebiotics is dependent on the species, which is a valuable characteristic for modulating the gut microbiota through the use of specialised prebiotics (Bielecka et al., 2002; Biedrzycka and Bielecka, 2004). The β-fructofuranosidase enzyme derived from *Bifidobacterium adolescentis* G1 exhibits a preference for fructooligomers over inulin as its substrate. This preference is also observed in *B. bifidum*. In contrast, it has been observed that *B. longum* and *B.animalis* possess the ability to hydrolyze a diverse array of FOS and XOS, including those derived from inulin (Bruno et al., 2002). According to a study conducted by Bruno et al. (2002), food products contain the greatest concentration of viable *bifidobacteria*. Likewise, the efficacy of *B. longum* supplemented with FOS is greater in curd. According to Martinez-Villaluenga and Gomez (2007), the probiotic *B. lactis* possesses the enzymes β-glucosidase and β -fructofuranosidase, which enable it to metabolise oligosaccharides present in fermented milk. This metabolic activity promotes the growth and metabolism of the probiotic organism. The therapeutic potentials and health benefits of different probiotics, prebiotics, and synbiotics are presented in **Table 4**.

**Table 4:** Therapeutic potential and health benefits of probiotics, prebiotics and synbiotics.

Biotic types	Sources	Diseases	Health effects	Mechanism of action	References
**Probiotics**	*Lactobacillus acidophilus* and *Bifidobacterium infantis*	Intestinal infections	Inhibition of *Staphylococcus aureus*, *Salmonella typhimurium*, *Yersinia enterocolitica, Clostridium perfringens* and *Aeromonas hydrophila*	Production of organic acids, bacteriocins and other primary metabolites, such as hydrogen peroxide, carbon dioxide and diacetyl	Shahani and Chandan, 1979; Laroia and Martin, 1990; Mishra and Lambert, 1996; Van der Meer and Bovee-Oudenhoven, 1998
	*L. casei, L. acidophilus* and *B. bifidum*	Immune enhancement	Data not available	Enhancement in non-specific (e.g. phagocyte function, NK cell activity) and specific (e.g. antibody and cytokine production) host immune responses	Kaila et al., 1992; Schiffrin et al., 1995; Gill, 1998
	*L. acidophilus*, *S. thermophilus, B. longum*, *L.* rhamnosus GG and *B. bifidum*	Diarrhoeal infections	Inhibitions of *Escherichia coli, Salmonella, Shigella, Clostridium difficile* and rotavirus	Production of organic acids, bacteriocins, hydrogen peroxide, carbon dioxide and diacetyl	Merson et al., 1976; Barefoot and Klaenhammer, 1983; Tojo et al., 1987; Oksanen et al., 1990; Siitonen et al., 1990; Hilton et al., 1997.
	*B. longum*, L. *casei Shirota*, L*. acidophilus, Bifidobaterium* spp. and L*. rhamnosus* GG	Cancer	Inhibition of tumour formation and proliferation	Inhibition of carcinogens and procarcinogens, bacteria converting procarcinogens to carcinogens, immune system activation, and reduced faecal enzyme levels	Lidbeck et al., 1991; Reddy and Rivenson, 1993; Goldin et al., 1996; McIntosh, 1996.
	*L. acidophilus*	Hypercholesterolaemia	Reduction of cholesterol levels	Assimilation of cholesterol and deconjugation of bile salts	Gilliland and Speck, 1977; Buck and Gilliland, 1994.
	*L. acidophilus, B. angulatum, B. breve, B. bifidum* and *B. longum*	Lactose intolerance	Utilisation of lactose	Production of β-D-galactosidase which hydrolyzes lactose	Kilara and Shahani, 1976; Hughes and Hoover, 1995.
	*L. acidophilus* and *Bifidobacterium* spp. Production of	Reduction of peptic ulcer, gastro-oesophageal reflux, non-ulcer dyspepsia and gastric cancer	Inhibition of *Helicobacter pylori*	Lactic and acetic acids, bacteriocins etc	Berrada et al., 1991; Lambert and Hull, 1996; Lankaputhra et al., 1996.
	*L. rhamnosus* GG	Food allergy	Help to relieve intestinal inflammation and hypersensitivity reactions in infants with food allergies	Hydrolyse the complex casein to smaller peptides and amino acids and hence decrease the proliferation of mitogen-induced human lymphocytes	(Sutas et al., 1996; Majamaa and Isolauri, 1997)
**Prebiotics**	Inulin from chicory roots	-	-	Stimulate the growth of *Bifidobacterium*	Gibson et al., 1995.
	Neosugar	–	-	Metabolised by the resident microbes in the colon including *bifidobacteria, Enterococcus faecalis, E. faecium, Bacteroides vulgates*, etc	Desai, 2008; Guarino et al., 2020.
	Isomalto-oligosaccharides (IMO) from miso, soy sauce and honey	–	Local and systemic Th-1-like immune response and regulation of immune function, balancing the dysbiosis of gut microbiota	*Bifidobacterium* and the *Bacteroides* groups can utilise IMO	Kohmoto et al., 1988; Wang et al., 2014.
	Xylooligosaccharides (XOS) from fruits, bamboo shoots, vegetables, honey, etc.	–	-	*B. adolescentis* utilizes xylobiose and xylotriose, whereas *L. lactis, L. rhamnosus* and *L. plantarum* utilise oat β-glucooligosaccharides	Okazaki et al., 1990.
**Synbiotics**	Food products containing *B. animalis* and amylose cornstarch	–	-	Promote the growth of *bifidobacteria*	Bruno et al., 2002.
	Curd containing *B. longum* and fructooligosaccharide (FOS)	–	Decrease cardiovascular risk factors, metabolic syndrome prevalence and markers of insulin resistance in elderly patients	Promote the growth of *B. longum*	Hughes and Hoover, 1995; Linares et al., 2017; Cicero et al., 2021.
	Oral synbiotic preparation containing *L. plantarum* and FOS	Sepsis in early infancy	Significant reduction in sepsis and lower respiratory tract infections	Promotes growth of *L. plantarum* ATCC202195	Panigrahi et al., 2017.
	Synbiotics containing five probiotics *(L. plantarum, L. delbrueckii* spp. *bulgaricus, L. acidophilus, L. rhamnosus, Bifidobacterium bifidum*) and inulin	–	-	Adult subjects with NASH (non-alcoholic s*teatohepatiti*s) demonstrated a significant reduction of IHTG (intrahepatic triacylglycerol)	Wong et al., 2013.
	Synbiotic products containing *L. rhamnosus, Bifidobacterium lactis*, inulin and oligofructose	Hepatic conditions	-	Increased level of intestinal IgA, reduced blood cholesterol levels and lower blood pressure	Pathmakanthan et al., 2002; Perez-Conesa et al., 2006.
	*L. rhamnosus* CGMCC1.3724 and inulin	Obesity	Weight loss	Reduction in leptin increase in Lachnospiraceae	Sanchez et al., 2014.
	*L. acidophilus, L. rhamnosus, B. bifidum, B. longum, E. faecium* and FOS	Obesity	Changes in anthropometric measurements	Decrease in TC, LDL-C and total oxidative stress serum levels	Ipar et al., 2015.
	*L. sporogenes* and inulin	Type 2 diabetes	-	Significant reduction in serum insulin levels and homeostatic model assessment cell function	Tajadadi-Ebrahimi et al., 2014.
	*L. casei, L. rhamnosus, S. thermophilus, B. breve, L. acidophilus, B. longum, L. bulgaricus* and FOS	Insulin resistance syndrome	The levels of fasting blood sugar and insulin resistance improved significantly	-	Eslamparast et al., 2014.
	*L. rhamnosus* GG, *B. lactis* Bb12 and inulin	Cancer	Increase in probiotics in stools and decrease in *Clostridium perfringens* led to increase in the IL2 in polypectomised patients	Increased production of interferon-ϒ	Safavi et al., 2013.

### 8.1 Synbiotics in ulcerative colitis

Synbiotics are products that combine probiotics and prebiotics, resulting in a synergistic interaction. This concept was introduced to address challenges in probiotic survival, especially during transit through the upper gastrointestinal tract. The use of a synbiotic enhances probiotic colonisation efficacy and facilitates the proliferation of probiotic strains (Pandey et al., 2015).

**Figure 4:**
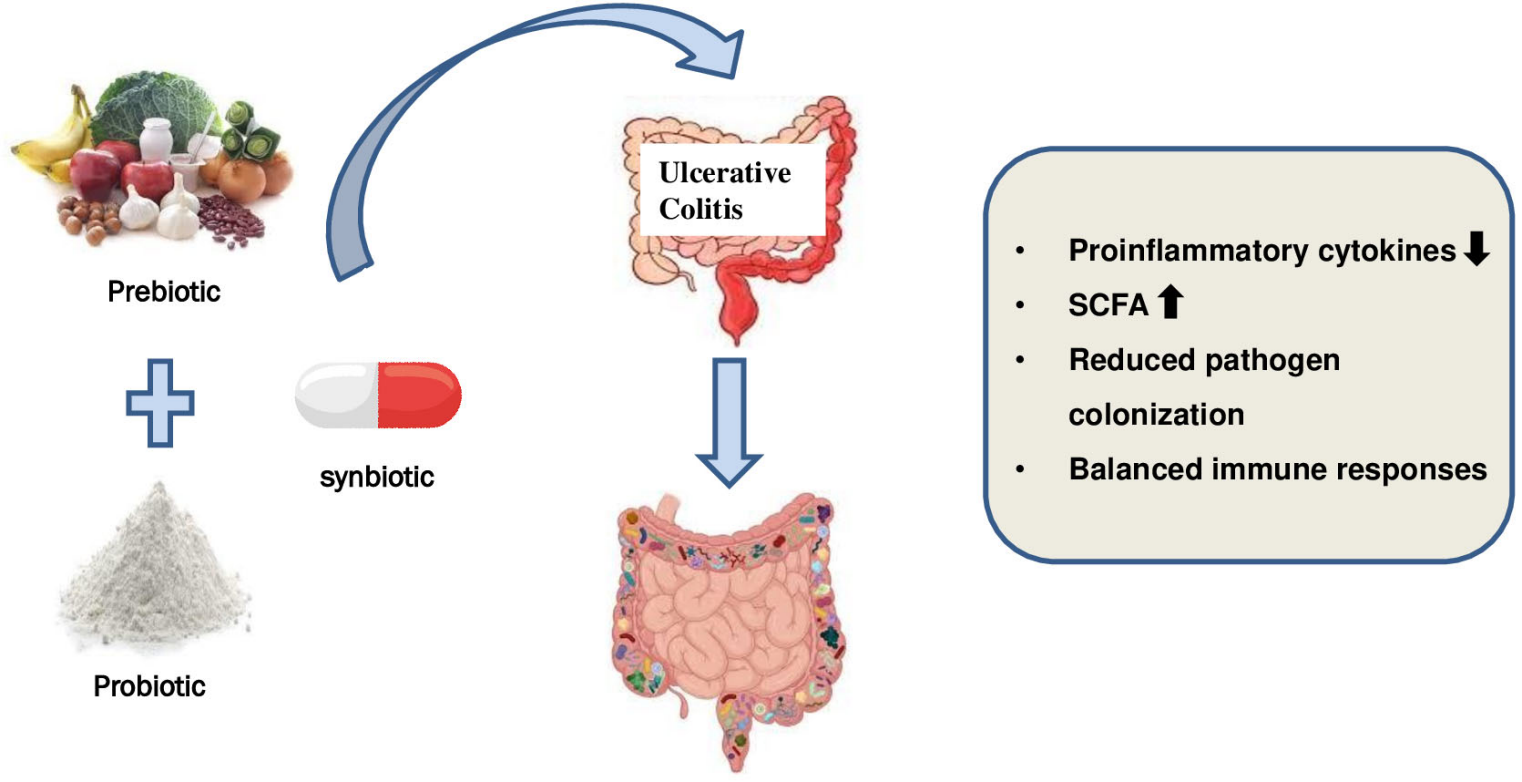
Use of synbiotics for ulcerative colitis treatment.

There is a limited body of research examining the impact of a synbiotic supplement on individuals diagnosed with UC. **Table 5** presents a compilation of the studies that have been cited most frequently and illustrated in **Figure 4**. Wong et al. (2022) investigated the impact of prebiotic mixtures, probiotic mixtures, and synbiotics on colitis in a murine model. The findings of the study indicated that the administration of synbiotic treatment had a protective effect on the structure of the colon, as evidenced by the preservation of its integrity. Furthermore, there was an observed increase in the expression of occludin, a protein involved in maintaining the tight junctions between cells, which is indicative of improved barrier function. Additionally, the treatment was found to be effective in reducing the infiltration of cells into the colon and resulted in alterations to the gut microbiome, enhancements in colonic integrity, and the suppression of markers associated with inflammation. Although the existing studies have yielded promising results thus far, it is important to note that the methodologies employed often exhibit inconsistencies, lack proper description, and/or suffer from inadequate study design. Additionally, it is worth mentioning that the majority of cases have been characterised by a notably limited number of registered patients.

**Table 5:** Summary of the most relevant studies involving synbiotics treatments in UC.

Sr. No.	Synbiotic	Outcome Results	Reference
1	*Bifidobacterium longum* plus inulin-oligofructose Treatment time: one month	Sigmoidoscopy scores ↓ β defensins 2, 3, and 4 ↓ CRP TNF-α ↓; IL-1β↓; IgA and IgG production↔	Furrie, 2005.
2	*Bifidobacterium longum* plus *psyllium* Treatment time: 4 weeks	CRP ↓ IBDQ (total, bowel, systemic, emotional, and social functional scores) ↑	Fujimori et al., 2009.
3	*Lactobacillus Paracasei* B 20160 + XOS Treatment time: 8 weeks	Serum IL-6, IL-8 ↓ Serum TNF-α, IL-1-β ↔ PBMC., IL-8 ↓	Federico et al. (2009)
4	*Bifidobacterium* breve strain Yakult plus galactooligosaccharides Treatment time: one-year	MPO ↓ Bacteroidaceae ↓ fecal pH ↓ clinical status: improved	Ishikawa et al., 2011.
5	*Lactobacillus acidophilus* LA-5^®^, *Lactobacillus delbrueckii* subsp. *bulgaricus* LBY-27, *Bifidobacterium animalis* subsp. *lactis* BB-12^®^ and *Streptococcus thermophilus* STY-31™ plus oligofructose Treatment time: One month	Microflora spectrum ↔	Ahmed et al., 2013.
6	*Streptococcus faecalis* T-110 JPC, *Clostridium butyricum* TO-A, *Bacillus mesentricus* TO-A JPC, *Lactobacillus sporogenes* plus prebiotic Treatment time: 3 months	Severity score ↓ Steroid intake ↓ Relapse during follow-up (3 months) ↓ Duration of remission ↑	Malathi et al., 2019.
7	A symbiotic which concluded six probiotics: *Enterococcus faecium*, *Lactobacillus plantarum*, *Streptococcus thermophilus*, *Bifidobacterium lactis*, *Lactobacillus acidophilus, Bifidobacterium longum* and fructooligosaccharide	The change in the CRP and sedimentation values had a statistically significant decrease in the synbiotic group (P = 0.003). The improvement in clinical activity was significantly higher in the synbiotic group (p < 0.05)	Kamarli et al., 2019.
8	*Lactobacillus plantarum* LP90 and Soluble dietary fibre obtained from *Lentinula edodes* by-products	Alleviated colitis	Xue et al., 2023.
9	*Bifidobacterium infantis and Bifidobacterium longum* and Equal parts FOS, GOS and XOS	Increased diversity of the microbiome and be associated with more SCFAs, and less gut inflammation	Ivanovska et al., 2017.
10	*L. paracasei* and *Opuntia humifusa* extract (mucilage + pectin)	Effective (greater abundance of L. paracasei in fecal microbial analysis, lower serum corticosterone levels, lower TNF-α levels in the colon tissue	Seong et al., 2020.
11	VSL#3 and Yacon (6% FOS + inulin)	Preservation of intestinal architecture, improve intestinal integrity, increased expression of antioxidant enzymes and concentration of organic acids	Dos Santos Cruz et al., 2020.
12	*Lactobacillus acidophilus, L*. *Rhamnosus, Bifidobacterium lactis* and Inulin	Increased the proportion of helpful bacteria and regulated the balance of intestinal microbiota, reduced the degree of inflammation in acute colitis mice	Wang et al., 2019.
13	LGG and Tagatose	Effective (gut microbiota composition recovered from the dysbiosis caused by DSS treatment)	Son et al., 2019.
14	*L. Casey* 01 and Oligofructose-enriched inulin	Assessment of colonic damage, inflammation scoring, MPO and microbiological studies are done and its effects on the UC	Ivanovska et al., 2017.
15	*Lactobacillus casei, Lactobacillus acidophilus, Lactobacillus rhamnosus, Lactobacillus bulgaricus, Bifidobacterium breve, Bifidobacterium longum, Streptococcus thermophiles* and FOS	Mitigated symptoms in patients with UC and suggested to use pre probiotics in the standard treatment, particularly in those with more than five years of the disease	Amiriani et al., 2020.
16	Food products containing *B. animalis* and amylose corn starch Data not available Data not available	*L. plantarum* utilise oat β-glucooligosaccharides to Promote the growth of *bifidobacteria*	Bruno et al., 2002.
17	Curd containing *B. longum* and fructooligosaccharide (FOS)	Promote the growth of *B. longum*	Hughes and Hoover, 1995; Linares et al., 2017; Cicero et al., 2021
18	Oral synbiotic preparation containing *L. plantarum* and FOS	Promotes growth of *L. plantarum* ATCC202195	Panigrahi et al., 2017.
19	Synbiotics containing five probiotics *(L. plantarum, L. delbrueckii* spp. *bulgaricus, L. acidophilus, L. rhamnosus, Bifidobacterium bifidum*) and inulin	Adult subjects with NASH (non-alcoholic *steatohepatitis*) demonstrated a significant reduction of IHTG (intrahepatic triacylglycerol)	Wong et al., 2013.
20	Synbiotic product containing *L. rhamnosus, Bifidobacterium lactis*, inulin and oligofructose	Increased level of intestinal IgA, reduced blood cholesterol levels and lower blood pressure	Pathmakanthan et al., 2002; Perez-Conesa et al., 2006.
21	*L. rhamnosus* CGMCC1.3724 and inulin	Reduction in leptin increase in Lachnospiraceae	Sanchez et al., 2014.
22	*L. acidophilus, L. rhamnosus, B. bifidum, B. longum, E. faecium* and FOS	Decrease in TC, LDL-C and total oxidative stress serum levels	Ipar et al., 2015.
23	*L. sporogenes* and inulin	Significant reduction in serum insulin levels and homeostatic model assessment cell function	Tajadadi-Ebrahimi et al., 2014.
24	*L. casei, L. rhamnosus, S. thermophilus, B. breve, L. acidophilus, B. longum, L. bulgaricus* and FOS	The levels of fasting blood sugar and insulin resistance improved significantly	Eslamparast et al., 2014.
25	*L. plantarum* La-5, *B. animalis* subsp. *lactisBB-12* and dietary fibres	Improvement in the IBS score and satisfaction in bowel movement reported	Smid et al., 2016.
26	*L. rhamnosus* GG, *B. lactis* Bb12 and inulin	Increased production of interferon-ϒ	Safavi et al., 2013.

Increase, ↑; decrease, ↓; equivalent; ↔.

## 9 Safety and considerations in ulcerative colitis treatment

When it comes to treating ulcerative colitis, ensuring safety and considering various factors are of paramount importance. Here are some key safety considerations to keep in mind:

### 9.1 Medical supervision

Ulcerative colitis treatment should always be conducted under the guidance and supervision of a qualified healthcare professional. They will assess the severity of the condition, consider individual patient factors, and prescribe appropriate medications or therapies (Kerner et al., 2014).

### 9.2 Monitoring and follow-up

Regular monitoring is crucial to evaluating the effectiveness of the treatment and assessing any potential side effects or complications. Patients should attend follow-up appointments as scheduled and communicate any changes in symptoms or concerns to their healthcare provider (Click and Regueiro, 2019).

### 9.3 Medication safety

Patients need to follow the prescribed medication regimen diligently. They should be aware of potential side effects and know when to seek medical attention. Certain medications used in ulcerative colitis treatment, such as immunosuppressants or biologics, may require additional precautions and monitoring due to their impact on the immune system (Click and Regueiro, 2019).

### 9.4 Individualised approach

Each patient with ulcerative colitis is exceptional, and management plans should be designed to meet their special needs. Factors such as age, disease severity, comorbidities, and medication tolerance should be taken into consideration when developing a personalised treatment strategy (Kerner et al., 2014).

### 9.5 Lifestyle modifications

In addition to medication, lifestyle changes can play a major role in managing ulcerative colitis. These may include dietary changes, stress management techniques, regular exercise, and getting sufficient rest. Patients should work with healthcare professionals, such as dietitians and psychologists, to make appropriate lifestyle adjustments (Click and Regueiro, 2019).

### 9.6 Risk-benefit assessment

Every treatment decision involves a careful evaluation of the potential risks and benefits. Patients and healthcare providers should discuss the expected outcomes, potential side effects, and long-term implications of various treatment options. This shared decision-making process ensures that the chosen treatment aligns with the patient’s goals and preferences (Kerner et al., 2014).

### 9.7 Complementary and alternative therapies

If considering complementary or alternative therapies, patients should consult with their healthcare provider to ensure safety and assess potential interactions with conventional treatments. It is important to be cautious of unproven or unsupported therapies that may promise a cure without scientific evidence (Click and Regueiro, 2019).

### 9.8 Patient education and support

Patient education about their condition, treatment options, and self-care is essential. Ulcerative colitis patients can benefit from educational resources and support groups. In conclusion, ulcerative colitis treatment must prioritise safety and individual factors. Patients can manage their condition while minimising risks and maximising treatment benefits with medical supervision, regular monitoring, personalised approaches, and informed decision-making (Kerner et al., 2014).

## 10 Future perspectives and research

UC significantly impacts individuals’ health and quality of life, with treatment options including aminosalicylates, corticosteroids, immunomodulators, biological therapies, and surgical interventions. However, some patients have not responded well to these treatments, leading to prolonged suffering and reduced quality of life. Alternative therapies like experimental drugs are considered, but their effectiveness remains uncertain and comes with risks and complications. As we gain more understanding of the pathophysiology of UC and its relationship with the microbiome, there will be a rise in research trials to determine the efficacy of emerging treatments. Scholars are currently investigating “next-generation” probiotics, such as *Clostridium clusters* IV, XIVa, and XVIII*, Bacteroides uniformis, Bacteroides fragilis, Akkermansia muciniphila, Eubacterium hallii*, and *Faecalibacterium prausnitzii* as potential alternatives.

Collaborative engagement between patients and healthcare providers is crucial for determining the most suitable treatment approach. Emerging treatments like prebiotics, probiotics, and synbiotics have gained attention for their potential therapeutic benefits in improving gut health and treating various conditions. For patients with IBD, traditional medications may not always work for every patient, and their side effects can be severe. In clinical practice, incorporating probiotics, prebiotics, and synbiotics holds great promise for transforming the approach to managing ulcerative colitis, offering patients a safer and more focused alternative to conventional therapeutic interventions. This will facilitate our understanding of the interplay between human physiological processes and the microbiome.

## 11 Discussion

The search for a definitive solution for UC continues to be a persistent effort, with existing therapeutic interventions primarily focused on maintaining a state of remission. In the management of this condition, conventional therapeutic approaches encompass the utilisation of aminosalicylates, corticosteroids, immunomodulators, and biological therapies. These interventions are employed with the objectives of attaining remission, averting relapses, and facilitating the restoration of mucosal integrity. Current therapeutic approaches encompass the use of monoclonal antibodies that specifically target proinflammatory cytokines, adhesion molecules, and T-cell activation, as well as anti-inflammatory cytokines like IL10 and TGF-. However, the administration of these medications can lead to various adverse effects, both at the systemic and local levels. These effects may include headaches, nausea, abdominal pain, loss of appetite, vomiting, and the development of a rash. The use of corticosteroids for an extended period is discouraged due to the potential increase in susceptibility to infections (Mowat et al., 2011). In contemporary times, functional food is increasingly recognised for its dual capacity to ensure nutritional security and confer health benefits on the consumer (Mijan and Lim, 2018). Promising advancements have been observed in the utilisation of probiotics, prebiotics, and synbiotics for the management of UC. Bacterial strains, such as *Lactobacillus* and *Bifidobacterium* species, have been linked to beneficial outcomes concerning symptom management and the maintenance of remission in individuals with a diagnosis of UC. By encouraging the growth of advantageous microorganisms and producing anti-inflammatory fatty acids, prebiotics supports gut health and digestion. Non-digestible fibres in foods like whole grains, garlic, and bananas support gut flora, lowering the risk of gastrointestinal conditions like irritable bowel syndrome and enhancing immune function. Prebiotic-rich foods are an excellent way to support digestive health and uphold general wellbeing through diet. The synergistic effects of prebiotics and probiotics allow for the effective management of UC (Fujimori et al., 2009). Therefore, it is imperative to conduct a comprehensive examination of these interventions to effectively mitigate any potential negative consequences and develop strategies for long-term supervision. The management of UC can be tailored to the individual by combining conventional treatments with probiotics, prebiotics, and synbiotics. The evaluated combination therapies demonstrate encouraging synergistic effects through the simultaneous targeting of various aspects of disease pathogenesis, including inflammation, immune dysregulation, and gut microbiota imbalance. Nevertheless, it is of utmost importance to conduct a thorough assessment of treatment interactions, contraindications, and individual patient variables to guarantee the safety and effectiveness of the intervention. It is imperative to undertake additional research, employing meticulously crafted clinical trials, to ascertain the most advantageous strains, compositions, and therapeutic regimens for probiotics, prebiotics, and synbiotics within the particular framework of UC. The aforementioned advancements provide empirical support for the capacity to enhance therapeutic outcomes, alleviate adverse effects, and establish innovative and well-tolerated strategies for managing UC. The current literature on probiotics and prebiotics in IBD is influenced by significant heterogeneity, with varying study designs, doses, and outcomes. The study populations varied, with some focusing on active disease patients and others on remission maintenance. Most studies enrolled small numbers of patients, limiting statistical power. The exact mechanisms of action of probiotics, prebiotics, and synbiotics are not yet fully understood. Insufficient evidence on probiotic dosages and immunological mechanisms is needed to establish health claims. The interaction between microbiota, host, and prebiotic components is also limited. Clinical trials and validation studies with larger sample sizes require understanding of these interactions. Limited published literature in manufacturing processes and formulation further needs improvement. (Astó et al., 2019)

In conclusion, the utilisation of probiotics, prebiotics, and synbiotics in the management of UC showcases a promising advancement with potential implications. These interventions present innovative strategies for manipulating the gut microbiota, reducing inflammation, and improving overall health. Additional investigation is required to determine the most effective utilisation and potential incorporation of these interventions into individualised therapeutic approaches for managing ulcerative colitis. Through the adoption of innovative approaches in the treatment of UC and the consideration of individual patient requirements, we have the potential to advance towards a more optimistic future for individuals afflicted by this complex inflammatory bowel disease.

## Author contributions

AJ: Conceptualization, Data curation, Writing – original draft. SJ: Conceptualization, Supervision, Validation, Writing – review & editing. SV: Writing – review & editing. AS: Writing – review & editing. BK: Writing – review & editing.
